# Erratum for Abdullah et al., “Whole genome-based genetic insights of *bla*_NDM_ producing clinical *E. coli* isolates in hospital settings of Pakistan”

**DOI:** 10.1128/spectrum.00113-24

**Published:** 2024-02-02

**Authors:** Sabahat Abdullah, Abdulrahman Almusallam, Mei Li, Muhammad Shahid Mahmood, Muhammad Ahmad Mushtaq, Nahla O. Eltai, Mark A. Toleman, Mashkoor Mohsin

Volume 11, no. 5, e00584-23, 2023, https://doi.org/10.1128/spectrum.00584-23. The article title should read as given in this erratum.

