# Supplementary material for: Whole genome-based genetic insights of bla NDM producing clinical E. coli isolates in hospital settings of Pakistant
Source: Microbiol Spectr. 2023 Sep 5;11(5):e00584-23. doi: 10.1128/spectrum.00584-23 (PMC10581159; doi:10.1128/spectrum.00584-23)
Supplement: Supplemental Table S1 — Antimicrobial susceptibility. [file spectrum.00584-23-s0001.docx]

**Supplementary Table S1. Antimicrobial susceptibility testing**

| **Isolate IDs** | **MEM** | **IPM** | **AMP** | **TEM** | **AMC** | **CTX** | **CAZ** | **AK** | **CIP** | **TET** | **TZP** | **SXT** | **TGC**  **(mg/L)** | **CST**  **(mg/L)** |
| --- | --- | --- | --- | --- | --- | --- | --- | --- | --- | --- | --- | --- | --- | --- |
| PK-5027 | R | R | R | R | R | R | R | S | R | R | R | R | 0.19 | 1 |
| PK-5034 | R | R | R | R | R | R | R | S | R | R | R | R | 0.25 | 0.5 |
| PK-5037 | R | R | R | R | S | R | R | R | R | R | R | R | 0.19 | 1 |
| PK-5052 | R | R | R | R | R | R | R | R | R | R | R | R | 0.75 | 1 |
| PK_5055 | R | R | R | R | R | R | R | S | R | R | R | R | 0.5 | 1 |
| PK-5068 | R | R | R | R | R | R | R | S | R | R | R | R | 0.25 | 2 |
| PK-5081 | R | R | R | R | R | R | R | R | R | R | R | R | 0.19 | 4 |
| PK-5092 | R | R | R | R | R | R | R | R | R | R | R | R | 0.19 | 0.5 |
| PK-5093 | R | R | R | R | R | R | R | S | R | R | R | R | 0.19 | 2 |
| PK-5095 | R | R | R | R | R | R | R | R | R | R | R | R | 0.5 | 1 |
| PK-5096 | R | R | R | R | S | R | R | R | R | R | R | R | 0.19 | 1 |
| PK-5099 | R | R | R | R | R | R | R | S | R | R | R | R | 0.25 | 1 |
| PK-5112 | R | R | R | R | R | R | R | R | R | R | R | R | 0.25 | 0.5 |
| PK-5116 | R | R | R | R | R | R | R | R | R | R | R | R | 0.25 | 2 |
| PK-5127 | R | R | R | R | R | R | R | S | R | R | R | R | 0.19 | 4 |
| PK-5136 | R | R | R | R | R | R | R | S | R | R | R | R | 0.19 | 1 |
| PK-5138 | R | R | R | R | S | R | R | S | R | R | R | R | 0.19 | 1 |
| PK-5140 | R | R | R | R | R | R | R | R | R | R | R | R | 0.5 | 1 |
| PK-5141 | R | R | R | R | R | R | R | R | R | R | R | R | 0.19 | 0.5 |
| PK-5144 | R | R | R | R | R | R | R | S | R | R | R | R | 0.19 | 0.5 |
| PK-5151 | R | R | R | R | R | R | R | R | R | R | R | R | 0.25 | 0.2 |
| PK-5152 | R | R | R | R | R | R | R | R | R | R | R | R | 0.25 | 2 |
| PK-5160 | R | R | R | R | R | R | R | R | R | R | R | R | 0.19 | 2 |
| PK-5171 | R | R | R | R | R | R | R | S | R | R | R | R | 0.19 | 1 |
| PK-5172 | R | R | R | R | R | R | R | R | R | R | R | R | 0.25 | 2 |
| PK-5176 | R | R | R | R | R | R | R | S | R | R | R | R | 0.125 | 0.5 |
| PK-5178 | R | R | R | R | R | R | R | R | R | R | R | R | 0.19 | 4 |
| PK-5179 | R | R | R | R | R | R | R | R | R | R | R | R | 0.19 | 1 |
| PK-5196 | R | R | R | R | R | R | R | R | R | R | R | R | 0.19 | 2 |
| PK-5198 | R | R | R | R | R | R | R | S | R | R | R | R | 0.25 | 0.5 |
| PK-5202 | R | R | R | R | R | R | R | S | R | R | R | R | 0.25 | 0.5 |
| PK-5209 | R | R | R | R | R | R | R | S | R | R | R | R | 0.25 | 2 |
| PK-5224 | R | R | R | R | R | R | R | S | R | R | R | R | 0.19 | 1 |
| PK-5238 | R | R | R | R | R | R | R | S | R | R | R | R | 0.125 | 2 |

Abbreviations: MEM, meropenem; IPM, imipenem; AMP, ampicillin; TEM, temocillin; AMC, amoxiclav; CTX, ceftriaxone; CAZ, ceftazidime; AK, amikacin; CIP, ciprofloxacin; TET, tetracycline; TZP, piperacillin-tazobactam; SXT, sulfamethoxazole/trimethoprim; TGC, tigecycline; CST, colistin.
