# Supplementary material for: Whole genome-based genetic insights of bla NDM producing clinical E. coli isolates in hospital settings of Pakistant
Source: Microbiol Spectr. 2023 Sep 5;11(5):e00584-23. doi: 10.1128/spectrum.00584-23 (PMC10581159; doi:10.1128/spectrum.00584-23)
Supplement: Supplemental Table S2 — Isolate descriptions. [file spectrum.00584-23-s0002.docx]

**Supplementary Table S2. Basic information of clinical *E. coli* isolates harboring *bla*_NDM_ gene**

| Isolate ID | Collection date | Hospital | Gender/Age | Specimen type | S1PFGE  (Size of plasmid) | Conjugation frequency |
| --- | --- | --- | --- | --- | --- | --- |
| PK-5027 | 19/9/2019 | Allied | Female/  58 | pus | 120kbps | 5.99x10^-8^ |
| PK-5034 | 13/11/2019 | Allied | Female/  49 | urine | 120kbps | 2.18x10^-6^ |
| PK-5037 | 14/11/2019 | DHQ | Female/  45 | pus | 90kbps | 6.73x10^-8^ |
| PK-5052 | 28/11/2019 | Allied | Male/  17 | urine | 150 kbps | 3.39x10^-7^ |
| PK-5055 | 29/11/2019 | Allied | Female/  43 | urine | 130 kbps | 4.67x10^-5^ |
| PK-5068 | 12/12/2019 | DHQ | Male/  40 | urine | 120 kbps | 2.81x10^-5^ |
| PK-5081 | 20/12/2019 | DHQ | Male/  68 | urine | 140 kbps | 1.77x10^-7^ |
| PK-5092 | 31/12/2019 | Allied | Female/  28 | urine | 130 kbps | 6.46x10^-8^ |
| PK-5093 | 1/1/2020 | Allied | Female/  49 | urine | 90 kbps | 5.33x10^-4^ |
| PK-5095 | 3/1/2020 | DHQ | Male/  34 | pus | 100 kbps | 6.11x10^-6^ |
| PK-5096 | 7/1/2020 | Allied | Female/  51 | urine | 120 kbps | 3.96x10^-8^ |
| PK-5099 | 10/1/2020 | DHQ | Male/  49 | urine | 40 kbps | 5.66x10^-7^ |
| PK-5112 | 24/1/2020 | DHQ | Female/  42 | urine | 150 kbps | 1.68x10^-6^ |
| PK-5116 | 30/1/2020 | Allied | Female/  51 | urine | 120 kbps | 2.32x10^-8^ |
| PK-5127 | 11/2/2020 | DHQ | Female/  45 | urine | 150 kbps | 3.95x10^-3^ |
| PK-5136 | 21/2/2020 | Allied | Female/  39 | urine | 140 kbps | 6.31x10^-7^ |
| PK-5138 | 21/2/2020 | Allied | Female/  68 | urine | 140 kbps | 5.62x10^-7^ |
| PK-5140 | 24/2/2020 | DHQ | Female/  42 | pus | 140 kbps | 1.95x10^-8^ |
| PK-5141 | 24/2/2020 | DHQ | Female/  36 | pus | 110 kbps | 6.45x10^-6^ |
| PK-5144 | 28/2/2020 | DHQ | Female/  52 | urine | 150 kbps | 5.06x10^-7^ |
| PK-5151 | 6/3/2020 | Allied | Female/  57 | urine | 190 kbps | 5.46x10^-4^ |
| PK-5152 | 6/3/2020 | DHQ | Female/  68 | urine | 150kbps | 3.77x10^-7^ |
| PK-5160 | 14/8/2020 | Allied | Female/  14 | urine | 250 kbps | 1.87x10^-6^ |
| PK-5171 | 27/8/2020 | Allied | Male/  63 | urine | 190 kbps | 2.78x10^-8^ |
| PK-5172 | 27/8/2020 | Allied | Female/  53 | urine | 130 kbps | 3.33x10^-3^ |
| PK-5176 | 2/9/2020 | Allied | Female/  48 | urine | 120kbps | 4.79x10^-3^ |
| PK-5178 | 8/9/2020 | Allied | Female/  38 | urine | 140 kbps | 1.68x10^-4^ |
| PK-5179 | 8/9/2020 | Allied | Female/  44 | urine | 150 kbps | 4.75x10^-6^ |
| PK-5196 | 20/10/2020 | Allied | Male/  52 | urine | 130 kbps | 6.06x10^-7^ |
| PK-5198 | 27/10/2020 | Allied | Female/  38 | pus | 140 kbps | 3.63x10^-8^ |
| PK-5202 | 5/11/2020 | DHQ | Female/  21 | urine | 110 kbps | 3.99x10^-6^ |
| PK-5209 | 20/11/2020 | DHQ | Male/  62 | urine | 130 kbps | 4.45x10^-7^ |
| PK-5224 | 10/12/2020 | Allied | Female/  55 | pus | 110kbps | 3.07x10^-6^ |
| PK-5238 | 21/12/2020 | DHQ | Male/  58 | urine | 110kbps | 5.35x10^-6^ |
